# Electric-double-layer *p–i–n* junctions in WSe_2_

**DOI:** 10.1038/s41598-020-69523-9

**Published:** 2020-07-30

**Authors:** Sara Fathipour, Paolo Paletti, Susan K. Fullerton-Shirey, Alan C. Seabaugh

**Affiliations:** 1 Department of Electrical Engineering, University of Notre Dame, Notre Dame, IN 46556 USA; 2 Department of Chemical and Petroleum Engineering, University of Pittsburgh, Pittsburgh, PA 15213 USA; 3 Department of Electrical and Computer Engineering, University of Pittsburgh, Pittsburgh, PA 15213 USA

**Keywords:** Electrical and electronic engineering, Electronic devices

## Abstract

While *p–n* homojunctions in two-dimensional transition metal dichalcogenide materials have been widely reported, few show an ideality factor that is constant over more than a decade in current. In this paper, electric double layer *p–i–n* junctions in WSe_2_ are shown with substantially constant ideality factors (2–3) over more than 3 orders of magnitude in current. These lateral junctions use the solid polymer, polyethylene oxide: cesium perchlorate (PEO:CsClO_4_), to induce degenerate electron and hole carrier densities at the device contacts to form the junction. These high carrier densities aid in reducing the contact resistance and enable the exponential current dependence on voltage to be measured at higher currents than prior reports. Transport measurements of these WSe_2_ *p–i–n* homojunctions in combination with COMSOL multiphysics simulations are used to quantify the ion distributions, the semiconductor charge distributions, and the simulated band diagram of these junctions, to allow applications to be more clearly considered.

## Introduction

Methods for forming *p–n* junctions in two-dimensional (2D) transition metal dichalcogenide (TMD) channels have been widely sought to enable electronic and optoelectronic applications^1^. Lateral TMD *p–n* junctions have been induced using a wide variety of approaches including buried gates (in WSe_2_ by Pospischil^2^, Baugher^3^, and Ross^4^, and in MoS_2_ by Sutar^5^), using combinations of buried gates and surface charge layers (in MoTe_2_ by Lim^6^), by ion gating using solid polymers (in MoTe_2_ by Xu^7^ and in WSe_2_ by Fathipour^8^), using ionic liquids (in WSe_2_ by Kozawa^9^ and Zhang^10,11^), by chemical doping (in MoS_2_ by Choi^12^ and Li^13^), and by thickness-dependent work-function engineering (in WSe_2_ by Xu^14^). A vertical *p–n* homojunction was demonstrated by Jin^15^ by transfer of Nb-doped, *p*-type MoSe_2_ onto transferred, undoped, *n*-type MoSe_2_ on SiO_2_ and an ideality approaching unity was achieved. In addition to these homojunction demonstrations, many 2D *p–n* heterojunctions have been demonstrated, as reviewed in Frisenda^16^, but few of these reports are ideal in the sense that the forward current increases exponentially with voltage for decades in current. The most ideal heterojunction reported is obtained in a transferred, vertical *p*-WSe_2_/*n*-InAs stack, by Chuang^17^, showing an ideality factor of 1.1 over approximately 4 orders of magnitude in current.

In homojunction TMD *p–n* junctions, the most ideal junctions have been achieved using buried gates^5^, or an ion-containing (solid polymers or ionic liquid) electrolytes biased to create an electric double layer (EDL) at the semiconductor surface. The double layer consists of a cation-electron or an anion-hole layer with a high capacitance density (e.g. 4 μF/cm^2^ as measured by Xu^7^). Once the double layers are formed, the ions are locked in place by cooling below a critical temperature. This method of doping in WSe_2_ has produced contact resistances as low as 3.4 and 1 kΩ μm (*n* and *p* respectively) and currents as high as 58 and 50 μA/μm at |*V*_*DS*_| = 2 V (*n* and *p* respectively)^18^. The method of forming *p-n* junctions by application of EDLs has its roots in the light emitting electrochemical cell as discussed by Pei^19^, Gao^20^, and Edman^21^. Our aim in this paper is to analyze the current–voltage (*I–V*) characteristics of the WSe_2_ *p–i–n* junction^7^ using the solid polymer, PEO:CsClO_4_. Through COMSOL multiphysics modeling we provide a quantitative physical understanding of the ion and carrier distributions in the electrolyte and channel. Simulation of EDL properties is of recent interest, as demonstrated by Ueda’s^22^ work using a drift–diffusion formalism. The doping method described in this paper has allowed demonstration of a homojunction WSe_2_ Esaki tunnel diode^23^.

## Results and discussion

**Figure 1:**
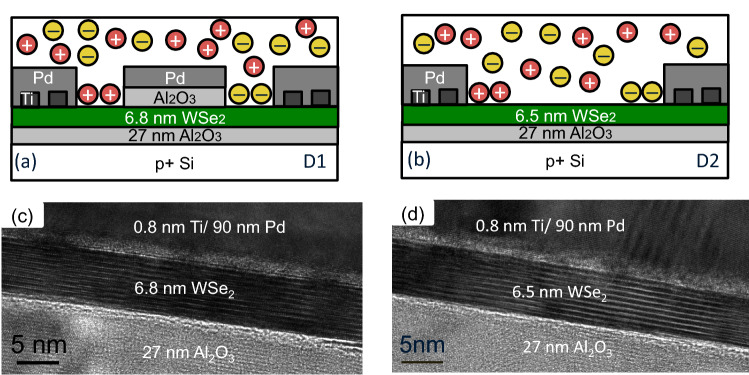
Schematic cross sections of two lateral WSe_2_ p–i–n junctions: (**a**) with top gate (5.3 nm Al_2_O_3_), (**b**) without top gate. (**c**) TEM image of contact region (Pd/Ti/WSe_2_) of device D1 and (**d**) device D2. The TEMs correspond to the same devices for which electrical measurements are reported.

Schematic cross sections for two *p–i–n* junctions are shown in Fig. 1a,b, respectively, in two different channel structures. Following the benchmarking of Sylvia^24^ for ultrascaled field-effect transistors (FETs), we focus on WSe_2_ as the channel material. Device D1 has a centered top gate with an Al_2_O_3_ thickness of 5.3 nm and device D2 has an open channel. Fabrication details are provided in the Methods section. The upper layer is the solid polymer electrolyte, PEO:CsClO_4_. The CsClO_4_ dissociates into cations, Cs^+^, and anions, ClO_4_^−^, as indicated by the circled + and − symbols in Fig. 1a,b. The metal contact to WSe_2_ consists of an electron beam deposition of Ti to partially cover the exfoliated WSe_2_ surface followed by Pd deposition. This leads to a dual work-function contact providing low Schottky barriers to both valence and conduction bands^18^. With a positive bias applied to the right contact with respect to the left contact, ions accumulate at the contacts as indicated in the schematic with the bulk of the PEO:CsClO_4_ remaining charge neutral. An EDL forms where the ions accumulate. Shown in Fig. 1c,d are transmission electron microscope (TEM) images of the contact region made after electrical measurements were completed. An interfacial layer at the scale of approximately 1 nm can be seen at the metal contact/WSe_2_ interface. This contact is formed by partially covering the WSe_2_ with Ti and then completing the metallization with Pd. For this reason, some transition layer can be expected. This dual work-function contact yielded contact resistances as low as 1 and 3.4 kΩ μm for *n* and *p* type contacts, respectively, comparable to the best reports for WSe_2_^18^. The physical attributes of the two devices are summarized in Table 1.

**Table 1:** Device structure parameters. The cool-down bias lists the drain and source biases, which are fixed during cooling to immobilize the ions.

	Device D1	Device D2	
Top gate length	1.5	No top gate	μm
WSe_2_ thickness	6.8	6.5	nm
Channel length	1.7	3.5	μm
Channel width	2	4	μm
Ti/Pd contacts	0.8/90	0.8/90	nm
Cool-down bias	*V*_*D*_ = − *V*_*S*_ = 1.5	*V*_*D*_ = − *V*_*S*_ = 2	V

To form the *p–i–n* junction, a positive bias is applied to the drain contact and a negative bias of the same magnitude is applied to the source contact, at room temperature. This accumulates negative ions at the drain contact and positive ions at the source contact, Fig. 1a,b. The ions then induce free carriers in the WSe_2_ of opposite sign, i.e. electrons at the source and holes at the drain. The structure D1 enforces an undoped region in the center of the channel because the top gate keeps ions out of the central channel region, while the open structure D2 has a central undoped channel due to the charge neutral electrolyte in the region between the electrodes. Once the ions are positioned along the channel, they are locked into place by cooling the device below the glass transition temperature of the PEO:CsClO_4_ (measured by Xu^7^ to be 240 K) while maintaining the biases on the contacts. Below the glass transition temperature, the ions are immobilized and do not respond to external biases and the device can be tested without ion reconfiguration.

**Figure 2:**
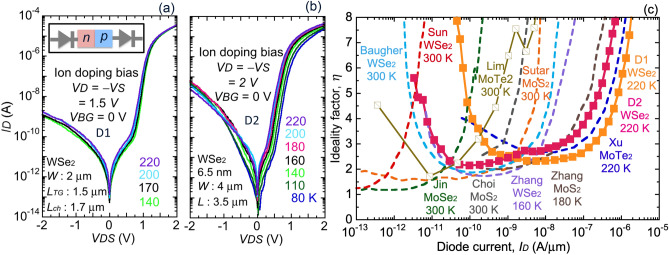
Temperature dependence of the *I*–*V* characteristics of WSe_2_ *p–i–n* junctions exhibiting exponential turn-on and clear rectification: (**a**) device D1 and (**b**) device D2. The inset in (**a**) is a reminder that the *p–i–n* junction is in series with an *n*-Schottky (left source contact) and a *p*-Schottky (right drain contact). (**c**) Comparison of ideality factor vs current per width for D1 and D2 vs. published TMD homojunction *p*–*n* diodes.

The transfer characteristic of the *p–i–n* diodes, D1 and D2, are shown in Fig. 2a,b, respectively. The *I–V* characteristics show a clear rectifying behavior with a forward to reverse current ratio of ~ 28,000 for D1 and ~ 2,000 for D2. More notable is the exponential dependence of the current on voltage over more than 4 orders of magnitude in both device geometries. The *p–i–n* junction is in series with metal/WSe_2_ Schottky contacts with an *n*-Schottky barrier on the left contact and a *p*-Schottky barrier on the right, Fig. 2a inset. Under forward bias, the two Schottky contacts are reverse-biased tunnel contacts resulting from the degenerate *n* and *p* carrier densities of the EDLs near the contact/channel edges. This series arrangement of Schottky barrier contacts means that the voltage across the junction will be somewhat less than the applied voltage.

The current in the forward-biased *p–n* junction, *I* = *I*_*O *_exp[*V*/*ηV*_*T*_], is predominantly controlled by the exponential factor *V*/*ηV*_*T*_ where *V* is the voltage across the *p–n* junction, *V*_*T*_ is the thermal voltage, *V*_*T*_ = *kT*/*q*, *η *is ideality factor, *I*_*O*_ is reverse saturation current, *k* is Boltzmann’s constant, *T* is temperature, and *q* is fundamental charge. The ideality factor of D1 and D2 can be extracted from the forward biased *I–V* characteristic using *η* = [(*kT*/*q*)ln(10)(dlog(*I*)/*dV*)]^−1^, which is valid when the applied voltage is predominantly dropped across the *p–n* junction. Figure 2c compares the ideality factor vs. current in D1 and D2 vs. published TMD *p–n* homojunctions. In Zhang^10^ and Sutar^5^ the width of the MoS_2_ junction was not specified and 4 μm is used. Choi’s^12^ report, on MoS_2_ using chemical doping, is an example where the ideality factor varies strongly with current, which is likely due to a series resistance. Sutar^5^ formed the *p–i–n* junction in MoS_2_ electrostatically by applying asymmetric biases to buried gates. The ideality factor in Sutar’s report is constant over 4 orders of magnitude in current with ideality less than approximately 2. The *p–i–n* junctions that extend to the highest currents in Fig. 2c were created by the formation of EDLs. Among these reports, Zhang^10,11^ used ionic liquids, while in the junctions of Xu^7^ and this work, PEO:CsClO_4_ was used. The WSe_2_ *p–i–n* junctions of this work exhibit substantially constant ideality factor vs. current, over 3 orders of magnitude; these lateral junctions show ideality factors ranging from 2 to 3. In contrast, the transferred MoSe_2_ homojunction of Jin^15^ exhibited nearly unity ideality, suggesting that trap-mediated generation/recombination^25^ is playing a role in lateral junctions. In the lateral *p–i–n* junction, the reverse leakage is also higher than the reverse saturation current, consistent with traps playing a significant role.

While it may appear that the D2 junction has a larger temperature dependence than D1, this is only because the measured temperature range is larger for D2. The temperature coefficient, Δ*I*/Δ*T* of the forward current is similar in the two junctions 1.4 (nA/μm)/K in D1 and 1.1 (nA/μm)/K in D2 (at 10 nA, normalized by the junction width *W*). The positive temperature coefficient in forward bias is opposite to what is expected from the exp(*qV*/*kT*) factor at fixed voltage. This is because the prefactor, *I*_*O*_*,* depends on the energy band gap, *E*_*G*_, making the full forward current proportional to exp[− (*E*_*G*_ − *qV*)/*kT*] as outlined by Sze^26^, and giving a positive temperature coefficient.

**Figure 3:**
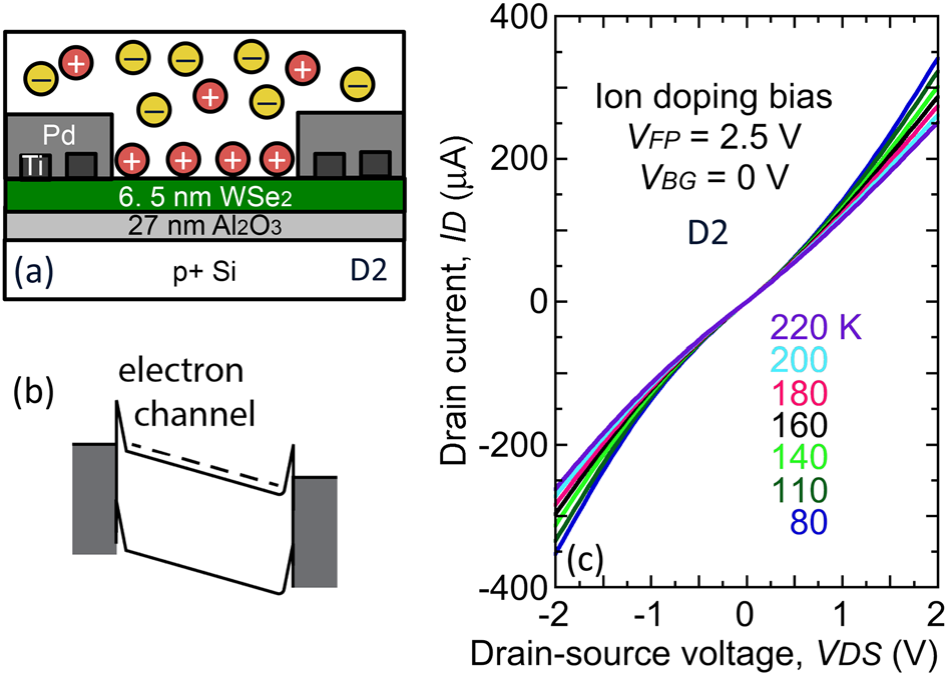
Unipolar doping of the WSe_2_ channel in device D2, used to measure Schottky contact temperature dependence. (**a**) Schematic cross section under side gate bias to accumulate positive ions on the channel, doping the channel *n*-type. (**b**) Corresponding band diagram for unipolar *n*-doping. (**c**) Symmetric, nonrectifying characteristics are obtained. To support the highest current measured in Fig. 2 requires less than ~ 0.4 V drop across the two contacts and access region.

The temperature coefficient of the Schottky contacts and access region can be separated out in the same device. To facilitate this, device D2 was cooled to below the glass transition temperature of PEO, with a 2.5 V side gate bias and 0 V on the source and drain contacts. Thus, positive Cs^+^ ions are driven onto the WSe_2_ surface, as indicated in Fig. 3a, to induce electrons in the channel, as described in the band diagram in Fig. 3b. Nonrectifying *I–V* characteristic were obtained as a result of this unipolar doping, Fig. 3c, and the temperature coefficient of the current is weakly negative.

A negative temperature coefficient is not readily explained from Schottky barrier transport, considering Schottky barrier lowering^27^ and thermionic field-emission^28^. A negative temperature coefficient is instead an indication of mobility degradation with temperature due to phonon scattering, as is also observed in Si metal–oxide–semiconductor FET inversion layers^29^. A negative temperature coefficient of the conductance has also been observed in WSe_2_ FETs using PEO:CsClO_4_^8^. The measured series resistance can be directly measured from Fig. 3c in the linear region below 1 V, where 8.7 kΩ is obtained, corresponding to a channel resistivity of 6.5 mΩ cm, which is reasonable for WSe_2_ mobility and sheet carrier density (100 cm^2^/Vs and 6 × 10^12^/cm^2^). Because both the *p–i–n* junction current and the series resistance increase with temperature, the voltage drop across the *p–i–n* junction decreases with temperature.

**Figure 4:**
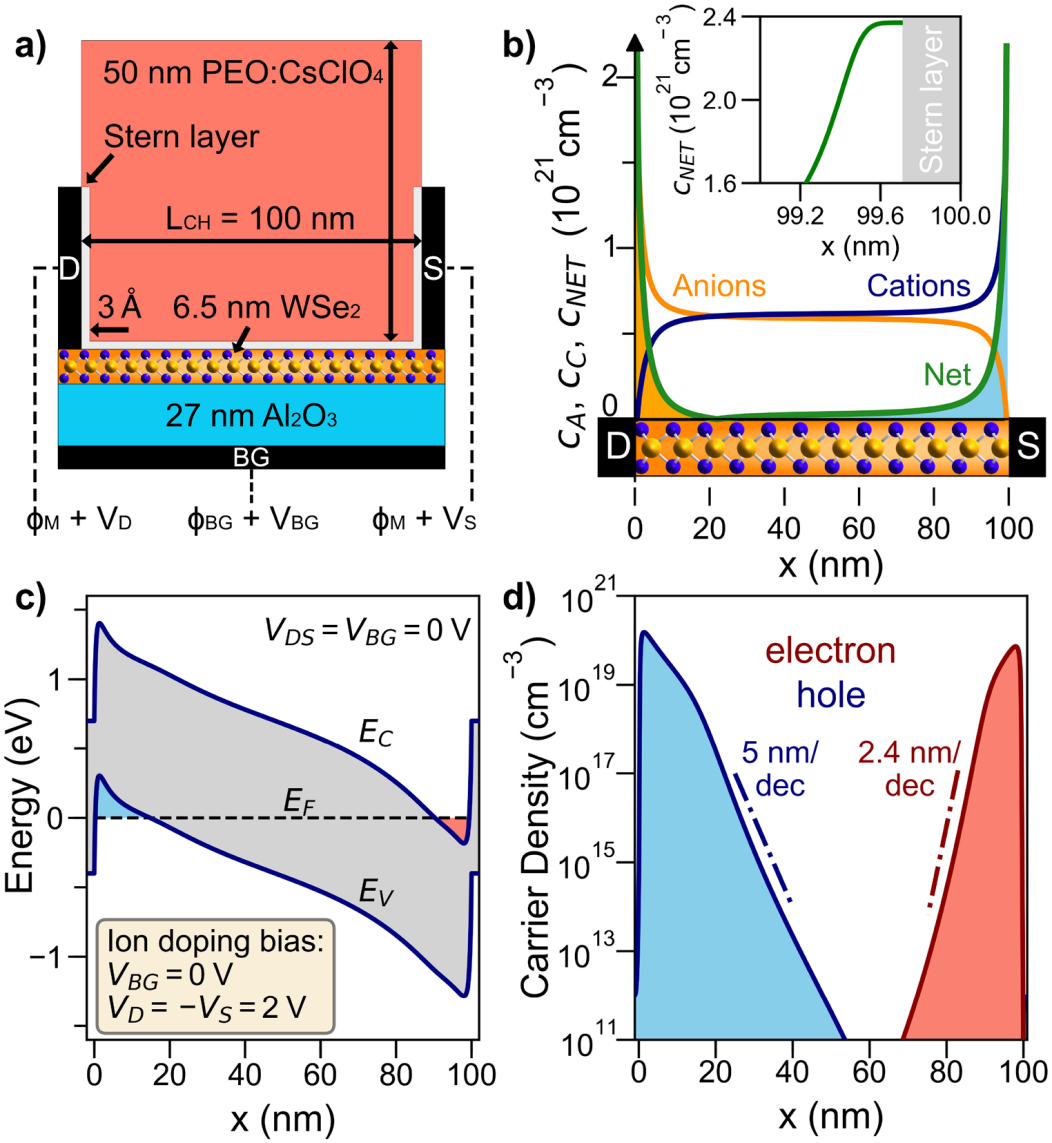
COMSOL simulations of the EDL *p–i–n* junction in WSe_2_. (**a**) Simulated device structure, representing a scaled-version of the fabricated device consisting of a 100 nm WSe_2_ channel and including source, drain, and backgate metal contacts, with PEO:CsClO_4_ on the surface. PEO:CsClO_4_ is modeled as a dielectric with *ε*_*PEO*_ = 7 and a concentration of 1,000 mol/L of monovalent ions. A thin, 0.3 nm vacuum layer at the metal/semiconductor interfaces with PEO:CsClO_4_ represents the effect of the Helmholtz layer. (**b**) Computed steady-state ion profile after a bias of *V*_*D*_ =  − *V*_*S*_ = 2 V is applied at the drain/source metal contacts. (**c**) Simulated band diagram along the channel length after the ion locking step at 220 K. (**d**) Charge density profile along the same cut showing an accumulation of image charges at the two ends of the channel, several orders of magnitude higher than that concentration in the middle of the channel.

COMSOL multiphysics simulations were performed to better understand the EDL junction formation. Given that this junction formation method is yielding the most ideal and high current homojunctions it is of interest to quantify the expected carrier densities and profiles. Figure 4a represents the simulated device structure for device D2, consisting of a 100 nm-long, 6.5 nm-thick WSe_2_ channel (*E*_*G*_ = 1.2 eV, *χ* = 3.9 eV) on 27 nm Al_2_O_3_. A shorter channel was used than in the experiments to reduce the simulation time while being sufficiently long to capture the electrostatic lengths of the ion distributions. On top of the semiconductor channel, there is a 50 nm layer of PEO electrolyte at a concentration of 1,000 mol/L of monovalent anions and cations. The presence of a Stern layer defining the separation between the ions in the solid polymer and the carriers in the semiconductor channel, is taken into account by inserting a 0.3 nm vacuum layer.

A modified Poisson–Nernst–Planck (MPNP) theory of electrodiffusion accounting for steric effects at large applied biases is applied to calculate the dynamics of ions within the electrolyte^23,30^. The system of equations consists of the Poisson's equation for the electrostatics and the modified Nernst–Planck equation for ion transport. The semi-classical transport of electrons and holes in the semiconductor is described by a drift-diffusion model. The drift-diffusion equations consist of the Poisson's equation for electrostatics, and the continuity equation for electrons and holes. Dirichlet boundary conditions are applied at the metal contacts (*φ*_*M*_ = 4.8 eV, *φ*_*BG*_ = 5.0 eV), while Neumann boundary conditions are used at the remaining boundaries. The semiconductor and the solid electrolyte domains are connected via an insulating interface so no charge transfer is enabled. Therefore, the two domains are coupled only electrostatically by means of the Poisson's equation.

The simulations follow the *p–i–n* junction formation protocol for device D2. First, at room temperature, the ion and semiconductor transport are solved self consistently, under an applied voltage of *V*_*D*_ =  − *V*_*S*_ = 2 V (*V*_*BG*_ = 0 V) to build-up the desired ion distribution in the electrolyte. The in-line electric field splits the positive and negative ions and as Fig. 4b suggests, ions accumulate at the two contacts and decay exponentially towards the middle of the channel. The high bulk ion concentrations resulting in a Debye screening length of order ~ 1 nm. To highlight the impact of the adopted MPNP, the inset of Fig. 4b shows the net ion concentration at the source extremity of the channel reaching a saturation value of *c*_*MAX*_ = 1/(*N*_*A*_* a*^3^) ≈ 3.94 mol/L (≈ 2.37 × 10^21^ cm^−3^), where *N*_*A*_ is Avogadro’s constant. The heuristic parameter, *a* = 0.75 nm, returns sheet charge density ~ 10^14^ cm^−2^ in accordance with experimental results^31^. This simple assessment reveals that the portion of the channel strongly affected by the presence of ions is mainly in close proximity to the source and drain contacts, hence it justifies our choice to simulate a scaled version of the fabricated device. The Al_2_O_3_ central gate in device D1 plays an insignificant role in the transport as the *p–i–n* junction is controlled by the high carrier density regions located within ~ 20 nm of the contacts. This is why devices D1 and D2 produce similar characteristics. The stabilized ion doping profile forms a *p–i–n* junction in the polymer, and the resulting net ion distribution mirrors the same junction at the surface of the underlying semiconductor channel.

With the computed steady-state room-temperature ion distribution, Poisson’s equation is then solved at 220 K under zero bias conditions using the net ion concentration as a fixed charge input. The accumulated anions (cations) near the drain (source) contact induce holes (electrons) in the underlying semiconductor layer. The equilibrium band diagram (*V*_*DS*_ = *V*_*BG*_ = 0 V) in Fig. 4c clearly shows that a barrier is formed due to the ion-separation, which is confirmed by the free carrier accumulation near the two ends of the semiconductor up to degenerate levels as shown in Fig. 4d. The band diagram also shows a graded profile due to the exponential decay in free carrier density. The graded profile reduces the abruptness of the junction profile, which is limited by the channel length linking the two highly-doped regions. The asymmetry in the hole and electron profiles is a consequence of the Schottky barriers at the metal contacts and the metal–oxide–semiconductor structure leading to a slight background accumulation of holes in the channel (~ 10^13^ cm^−3^). The carrier density slopes, 2.4 nm/decade for electrons and 4.1 nm/decade for holes, are highly abrupt relative to impurity dopant slopes, which are larger by a factor of two or more^32^.

## Conclusions

Lateral EDL WSe_2_ *p–i–n* junctions are demonstrated with substantially constant ideality factors over nearly 4 orders of magnitude using PEO:CsClO_4_ to accumulate electrons and holes at the channel contacts. The high carrier densities at the contacts lead to a low resistance of the Schottky barriers and a series resistance which is dominated by the channel access resistance. The lower series resistance of these structures enables observation of the exponential dependence of the forward current over a wider range than previous studies. COMSOL simulations reveal that degenerate carrier densities are induced with abrupt carrier profiles in the vicinity of the contacts. The junction formation method of this paper using PEO:CsClO_4_ and without a side gate achieves the highest currents with most ideal rectification properties reported to date in doping homojunctions in 2D materials.

In considering applications of this junction formation method, the requirement that the ions need to be positioned under bias and cooled to freeze them in place is undesirable and likely impractical. This freezing requirement can be lifted if a polymer with a higher glass transition temperature is used, as discussed by Kinder^33^, or if the EDL-induced junction can be frozen in place at room temperature via crosslinking using a thermally triggered polymerization, as demonstrated by Liang^34^. Another way to eliminate the need to freeze the ions in place is to make the ion positioning bias and the operating bias the same; in this case cooling is not required as the fixed forward bias holds the ions in place. This is used, for example, in *p–i–n* junctions formed by this technique and used as light-emitters^19,20^. However, if the terminal biases change, the ions will re-equilibrate and the emission characteristics can be expected to change accordingly.

Another question for this technology is scalability. There exist reports of carbon nanotubes (CNTs) with diameter of 2 nm coated and controllably electrolytically gated with PEO:LiClO_4_^35^, showing that there is no intrinsic limit to coating cylindrical structures at the 2 nm scale. InAs nanowires with 50 nm diameter have also been successfully coated and gated with PEO:LiClO_4_^36^. The electrolyte itself can also be scaled; ultrathin films of PEO have been spin coated down to 8 nm thickness, and exhibit well-behaved electrical properties^37^. However, to our knowledge there is no investigation of the viability of this doping method in highly-scaled VLSI (very large scale integration) geometries. Regarding the scaling of the TMD thickness, there are no fundamental impediments to the junction formation approach at the single monolayer thickness.

## Methods

Devices D1 and D2 were fabricated in separate process runs. The fabrication began with backside evaporation of Ti/Au (5/100 nm) on the unpolished side of a *p*^+^ Si wafer. Next, 27 nm of Al_2_O_3_ was deposited on the Si top surface by atomic layer deposition (ALD). Synthesized WSe_2_ flakes (from 2D Semiconductors Co. with 99.9995% purity) were exfoliated on the oxide using dicing tape (Semiconductor Equipment Corp. P/N 18074). The flakes were patterned for source and drain using electron beam lithography (EBL) followed by metal deposition of Ti/Pd (0.8 nm/90 nm) and lift off. Device D1 omitted the gate process. Device D2 used a TiOPc adhesion layer following by the ALD process of Park and Fathipour^38^. Finally, the top gate contacts were patterned using EBL, followed by thermal metal deposition of 90 nm Pd and lift off. The oxide was then etched from the access regions of the FET in buffered HF, using the top gate as an etch mask. The device structures of this paper were fabricated in the same process run reported in reference^18^; in that paper the transistor and contact properties were analyzed using side-gates to position the ions. In this paper we focus on the formation of *p–i–n* junctions in transistor geometries without the use of auxiliary gates.

The solid-polymer electrolyte preparation and materials have been previously detailed^18^. In short, PEO (95,000 g/mol) and CsClO_4_ were dissolved in anhydrous acetonitrile at a concentration of 1 wt%, and an ether oxygen to Cs^+^ molar ratio of 168:1. The solution was drop-cast in an Ar-filled glove box, < 0.1 ppm O_2_ and H_2_O, and then annealed for 3 min at 90 °C. Current–voltage measurements were made in a Cascade Microtech PLC50 vacuum probe station at 1.2 × 10^–6^ Torr. Devices that employ PEO:CsClO_4_ are stable and give reproducible electrical characteristics under testing over months, as long as the applied voltages are kept within the electrochemical window of the electrolyte (± 4 V) and wafers are stored between testing in an Ar or vacuum ambient to prevent water absorption of the PEO.
